# The fading self in space-disruption of default spatial representation across neurological disorders

**DOI:** 10.3389/fnsys.2025.1655500

**Published:** 2025-12-29

**Authors:** Ravinder Jerath, Varsha Malani

**Affiliations:** 1 Mind Body and Technologies, Augusta, GA, United States; 2 Master of Science, Northeastern University, Boston, MA, United States

**Keywords:** body schema, cognitive map, default mode network, DMN, default spatial representation, DSR, auditory hallucinations, schizophrenia

## Abstract

Neurological disorders stem from an intermingled change to self-in-space. While many of these disorders present as spatial deficits—contralateral neglect syndrome, for example—they manifest from the same etiology: disruption to the brain’s “default spatial representation” (DSR). DSR is a basic internally generated representation of space that delineates where the self is located in space—without attentional focus from an external drive. We review how pathologic disintegration of DSR is associated with anomalous activation and connectivity within distinct large-scale brain networks (e.g., the default mode network and a comprehensive attention-networked system), leading to a heterogeneous presentation of clinically assessed outcomes. The outcomes include psychogenic paralysis of limbs, left-side neglect, rectified sense of other locations, disorders of consciousness, symptoms related to autism spectrum disorder, Alzheimer’s disease, schizophrenia, and depersonalization/derealization disorder. By consolidating evidence from neuroimaging, lesion-symptom mapping, and computational assessment, we aim to reconceptualize these disorders not as separate and independent maladies, but as manifestations of a deeper, shared etiology, supporting a network-based assessment strategy for diagnosis and treatment that seeks to restore self-in-space.

## 1 Introduction

The brain’s intrinsic spatial canvas, the constitution for operation and existing within space, relies on a definitive sense of spatial orientation and awareness of where one’s body exists within it. Yet many neurological disorders involve disruptions to this critical awareness of being, resulting in permanent symptoms that go beyond sensory or motor dysfunction. (Penfield and Rasmussen, 1950). People deny they have a side, acknowledge they live somewhere else, and fail to see half of their visual field; yet somehow, they find themselves inundated in a fragmented reality that cannot be rendered in proper interpretation. To create sound diagnostic and treatment strategies, the commonality of such diverse yet connected conditions needs to become widely known.

One ideal disorder to explore in this assessment is contralateral neglect syndrome. This disorder occurs mostly after right hemisphere strokes; however, patients with neglect do not acknowledge, respond to, or even report stimuli presented in contralesional space (Heilman et al., 2012)—typically, the left hemispace—even when sensory and motor input/output abilities are intact. In fact, it is not a problem of vision; it is a problem of attendance and awareness (Driver and Mattingley, 1998). Therefore, a patient could only eat from one side of the plate—with sensory awareness around the plate still there—or shave half their face or run into left-sided walls. Its profoundly disability-inhibiting nature indicates how consistent realignment with self-in-space is necessary to function (Karnath, 2015).

In addition, specific brain regions neglect has been traditionally attributed to lesions in specific brain areas, most notably the right posterior parietal cortex (Rorden and Karnath, 2004). However, due to the varied symptoms of neglect and its potential overlap with lesions in various other cortical and subcortical areas (e.g., the frontal eye fields, thalamus, and basal ganglia) (Karnath et al., 2001), scholars have shifted their stance regarding its origin. More and more researchers view these deficits not as resultant from localized, regional failures, but instead, as failures generated by dysfunctional processing among large-scale, integrated brain networks. Yet, the integrative nature is important for relative future understanding.

Default spatial representation: We purport that many of these disorders come from failure around the brain’s “default spatial representation” (DSR). This tenet is paramount as it focuses on the brain’s default, flexible, ever-evolving internal reconstruction of a person’s place, orientation, and proportion in space relative to external environmental stimuli. This DSR exists as the primary “canvas”/"blueprint” along which all subsequent relative spatial processing, attention, and awareness happen. The DSR exists in the absence of externally based attentional drive, with preattentive “self” spatial consciousness occurring all the time.

Importantly, this DSR is likely aligned with the default mode network (DMN) composed of various regions (medial prefrontal cortex, posterior cingulate cortex, precuneus, angular gyrus) that are activated reliably when at rest, during self-referential processing, and internal thinking (Buckner et al., 2008). The DMN is believed to sustain a constant, internal representation of self and environment (Fox and Raichle, 2007). Therefore, we assert that an important function of the DMN—or a similarly associated network—is to uphold this DSR. In addition, the DSR interfaces with attention networks (e.g., dorsal attention network and ventral attention network), which facilitate overt attention to specific locations in space (Corbetta and Shulman, 2002), as well as the Global Neuronal Workspace (GNW), which specializes in transmitting information for conscious access (Dehaene and Changeux, 2011). For something to be consciously accessible and globally available from a specific space, it must acknowledge integration with the fundamental framework of DSR first.

This paper has utilized collective evidence through neuroimaging, lesion-symptom mapping, and computational modeling to produce a new re-conceptualization of a variety of neurocognitive disorders by proposing that, despite heterogeneous presentations, they are all linked by one significant deficit: failure of the “default spatial representation.” This will explain why these patients have difficulty with “fading” or misrepresentation of spatial awareness and how networked diagnostic and treatment solutions may emerge in the intervention to restore proper self-in-space awareness.

## 2 Contralateral neglect syndrome

A Default Spatial Representation Disruption Clinical Picture: Contralateral neglect syndrome is a fascinating yet debilitating constellation of symptoms. Individuals can neglect contralateral stimuli (i.e., left side after right-sided damage), resulting in personal neglect (neglect of one half of their body, e.g., putting on clothes but only for one arm), peripersonal neglect (neglect of objects within reach, e.g., only eating off the right side of the plate), or extrapersonal neglect (neglect of far-away objects, e.g., failing to see a car coming from the left) (Heilman et al., 2012). This can emerge as motor neglect (underuse of the contralesional side that is not due to primary motor direct weakness), sensory neglect (failure to respond to stimuli despite intact sensation), and representational neglect (neglect of the contralesional portion of an imagined image, e.g., claiming to recall only the right side of a known building when it is reported) (Bisiach and Luzzatti, 1978). The all-pervasive quality of the features makes this a complication for daily function and rehabilitation, thus making it an area of extensive research (Karnath, 2015).

Lesion Sites in Classical Lesions: The typical lesion sites associated when one thinks of contralateral neglect are lesions with the right inferior parietal lobule (IPL), particularly the temporoparietal junction (TPJ) and angular gyrus; the superior temporal gyrus (STG); the frontal eye fields (FEF) located in the prefrontal cortex; and subcortical areas, including the thalamus (pulvinar) (Sherman, 2014) and basal ganglia (putamen) (Karnath et al., 2001). Thus, these met sites show that neglect is not just an issue of the “parietal lobe,” but something much more complicated.

Network Dysfunction in Neglect: But why do different sites lead to equivalent ymptoms? Why is it a worse type of neglect that is more chronic when the right hemisphere is damaged as opposed to the left? This is because traditional models support a unitary attentional system (Mesulam, 1981, 1999).

We suggest that localized lesions offer a disruption to the coherence and balance of large-scale networks, as a whole, responsible for DSR. For example, neglect can arise from dual-task interference between the ventral attention network (VAN), hemispherically lateralized to the right and responsible for reorienting to salient stimuli (Seeley et al., 2007), and the dorsal attention network (DAN), responsible for top-down goal-directed attention (D’Esposito et al., 2003). Furthermore, the DMN, with its internal model function, plays a central role (Bzdok and Eickhoff, 2015). New resting-state fMRI work found that neglect patients have compromised networked functional and structural connectivity (Umar et al., 2023). Lesions to the right hemisphere disrupt internal structural integrity of white matter tracts among larger nodes of VAN and DMN, leading to reduced functional connectivity within such networks and interhemispheric imbalance in communication (Grefkes and Fink, 2014; Catani and de Thiebaut Schotten, 2008). For example, SLF between parietal and frontal regions is consistently involved (Bartolomeo and de Thiebaut Schotten, 2016). Such network compromise causes the “fading” or systematic erasure of contralesional space from the default spatial sketchpad of the brain. The right hemisphere’s responsibility for global spatial processing and maintenance of the general body-in-space schema positions it to facilitate the process, and maintaining a comprehensive body-in-space schema makes it particularly vulnerable to producing neglect when damaged (Mesulam, 1999; Sperry, 1961). The left side of space, no longer adequately represented in this intrinsic spatial model, effectively “fades” from awareness and action (Driver and Mattingley, 1998).

## 3 Other disorders of the default spatial representation

This section will discuss other disorders of interest, noting how each functions as a different form of dysfunction observed in “default spatial representation.”

### 3.1 Anosognosia

Impaired representation of self-body Anosognosia represents impressive but unfortunate symptoms whereby patients fail to acknowledge or, worse, deny their neurological deficit (e.g., paralysis and blindness). It occurs with contralateral neglect, where patients ignore half their physical body. Experiencing an inability to acknowledge its existence is common amongst patients post-right hemisphere stroke (Bisiach and Geminiani, 1991). Presently, we care to understand how the brain can deny acknowledgement of such an obvious deficit. Anosognosia is a failure to update the brain’s predictive model of what it is or what it can necessarily sense; as suggested previously, the DSR exists from the general mind awareness of what it can accomplish. For example, DSR relies upon a continuously updated, non-consciously available body schema to orient one’s sense of self across time epochs (Tsakiris, 2017). Thus, when it fails to update such status and instead postulates to the patient that their limb is functioning when it is not, they believe it and think their affected limb is still fine (Guterstam et al., 2015). fMRI studies implicate the right insular cortex (Craig, 2009), right temporoparietal junction, and frontoparietal network as critical for anosognosia (Fotopoulou and Tsakiris, 2017; Vallar and Calzolari, 2018). These regions are associated with integrating interoceptive signals (e.g., body sensations, states) and exteroceptive signals (i.e., signals from the outside world) as well as self-monitoring. When these regions fail in anosognosia, the information relative to the updating of such realities in DSR cannot happen, creating a new DSR that fails to incorporate new information but keeps the old, inaccurate information. Thus, patients can no longer align with what they feel on the inside or perceive from exogenously projected elements over transverse temporal consciousness for accurate self-representation.

### 3.2 Spatial disorientation and topographical disorientation

Confounded by accessing the internal cognitive map, patients with spatial disorientation cannot navigate familiar places, whereas those with topographical disorientation cannot identify previously known landmarks or learn new routes, despite retained basic visual perception abilities and general memory. Here, the acute concern is the breakdown of representational affording for spatial navigation. These disorders suggest the DSR’s ability to acquire a cognitive map/stabilize one over time has been compromised—inherently or allocentrically—for the outside world. The DSR creates a default template by which we subsequently encode and retrieve places over time (O’Keefe and Nadel, 1978). Recent literature implicates regions comprising or heavily connected to the DMN via functional connectivity, including retrosplenial cortex (RSC) (Vann et al., 2009), parahippocampal place area (PPA) (Maguire et al., 1998), and entorhinal cortex (Epstein et al., 2017). An inconsistent ability to maintain a connection between what one determines as their own movement to world-situated landmarks means that instead of an integrated spatial map that directs us (Maguire, 2001), we feel “lost in the mental map.”

### 3.3 Disorders of consciousness (coma, vegetative state, and minimally conscious state)

The inoperative spatial self is one of the most severe conditions where individuals experience a compromised or complete loss of objective awareness of themselves. Here, attention rests with determining the neural correlates of consciousness and differentiating between these disorders (Laureys et al., 2005). One prominent feature that connects these disorders is the disrupted integrity and functional connectivity of DMN across states (Boly et al., 2017; Crone et al., 2011). We propose that failing to maintain a coherent DSR across time diminishes one’s ability to consciously exist within themselves, which is critical for awareness throughout the lifespan. The pronounced disruption in DMN integrity and functional connectivity is an ongoing hallmark of these disorders (Boly et al., 2017; Crone et al., 2011). We believe that the inability to sustain a coherent DSR is central to the collapse of subjective experience and self-awareness. Without this fundamental spatial layout, the brain cannot construct a coherent “self-in-space” (Perani et al., 2011). New fMRI and EEG studies illustrate reduced DMN activity and compromised long-distance functional connectivity (e.g., between DMN nodes and frontoparietal networks) in DOC patients (Goldfine et al., 2011; Martini et al., 2019). The coherence of the DMN generally aligns with the consciousness level and outcome (Owen et al., 2006; Massimini et al., 2005; Rosanova et al., 2012). This has the implication that the ability of the DMN to maintain a continuous DSR is a basic requirement for conscious experience in itself (Schiff, 2010).

### 3.4 Autism spectrum disorder (ASD)

Atypical social and self-space ASD is defined by social communication deficiencies and restricted and repetitive behaviors. However, the challenge is determining what all of these symptoms mean neurologically. Consistent DMN connectivity and functional deficits characterize ASD (Buckner et al., 2008; Vercillo et al., 2020). We posit that a dysfunctional DMN contributes to a dysfunctional DSR, reflecting not only the self but also social spaces and boundaries between the self and others, as well. For example, the confirmation of hypo- and hyper-connectivity within the DMN is present in ASD, as well as in disrupted functional coupling between the DMN and other networks (e.g., salience network; Yeo et al., 2011). Such dysfunction promotes problems in perspective-taking, social cues, and interpersonal reactions, for at an extremely basic level, the most fundamental space where self exists and the self-other dyad is processed, is rendered dysfunctional.

### 3.5 Alzheimer’s disease and other dementias

Eroding spatial memory and self-problem: Dementias—especially Alzheimer’s disease (AD)—are related to abnormal memory and cognitive functions that develop over time, often resulting in dramatic spatial disorientation (Alzheimer’s Association, 2023). The problem is showcasing how these changes occur early on at the neurological level, but impact the most critical cognitive variables. The DMN is one of the first networks of the brain to be abnormally activated in AD, usually having its hubs filled with amyloid-beta plaques or tau tangles (Greicius et al., 2004; Buckner et al., 2009). We posit that the involvement of the DMN via creation of DSR underlies why individuals early on struggle with spatial disorientation, and why, over time, their coherent sense of identity fades. Longitudinal studies show that the disruption of the DMN occurs before overt cognitive symptoms manifest in AD (Sperling et al., 2011). A compromised DMN assists in how spatial memories are created/retained (a factor of DSR) and how a stable, consistent sense of self can occur, engendering a “fade” of sense of personal history and awareness of space (Raichle, 2015).

### 3.6 Schizophrenia: distorted reality and self-boundaries problem

Schizophrenia is associated with dramatic disruptions in thought and perception (hallucinations) and beliefs (delusions), manifesting as disruptions in self-identification. However, the challenge is pinpointing their neurological foundations (Frith, 2005). The DMN is often dissociated from functional tasks in individuals with schizophrenia and those diagnosed (Whitfield-Gabrieli and Ford, 2012; Garrity et al., 2007). We posit that such disconnects occur because they create a default space of reality that unknowingly dissociates internal constructs from external sentiment or confusion on what is an internalization and what is an external perception (Frith, 1992). For example, we see that hyperactive DMNs or dysfunction, dependent upon coupling with task-positive networks, can render hallucinations (confusing internal dialogue with external auditory contributions) or delusions of control (confusion over agency with regard to one’s own efforts; Palaniyappan and Liddle, 2012). Ultimately, this marks a major malfunction of the DSR to appropriately signify where self-boundaries versus engagement with others exist in their external world (Feinberg and Campbell, 2010; Northoff, 2016).

### 3.7 Depersonalization/derealization disorder: detachment from self and space problem

Depersonalization/derealization disorder consists of continuous or episodic detachment from one’s body/mental processes (Medford et al., 2005) or environment (derealization) (Sierra and Berrios, 2000). The primary problem is the perception of unreality. These phenomena speak directly to a compromised DSR subjectively. To feel as if one’s body or world is “unreal,” “dreamy,” or “faded” speaks of an incredible potential breakdown of the brain’s foundational, ever-present notion of self-in-space (Blanke and Metzinger, 2009). Furthermore, fMRI studies show altered connectivity patterns within the networks responsible for self-representation, emotional regulation, and interoceptive awareness, including the DMN salience network (Seeley et al., 2007) and functional fronto-limbic circuits (Lim et al., 2016; D’Souza et al., 2004). Ultimately, this reveals a decoupling from what should be an emotionally and sensory grounded DSR to facilitate subjective detachment and a sense of unreality.

## 4 Disruptions of DSR in neuropsychiatric and neurological disorders

Table 1 demonstrates some neuropsychiatric and neurological disorders that reflect the clinical relevance of DSR. For each condition, a network that is disproportionately impacted is presented on a connectomic scale. Additionally, the column to the right shows how such disconnections are resonant with failures of DSR components—either due to lack of affective coherence, social decoding failures, and disruptions in embodied control:

**Table 1:** Key disorders, affected networks, and dsr component disruptions.

Disorder	Primary network(s) affected	Disrupted DSR components (examples)
Autism spectrum disorder (ASD)	Default mode network (DMN) hypoconnectivity in social-cognitive hubs; atypical salience network (SN) signaling	Dysfunction in social understanding (i.e., theory of mind deficits associated with DMN dysfunction); problems blending emotional information (affective coherence) with the self; stereotyped or unusual sensorimotor feedback
Schizophrenia	Dysregulated DMN (hyperconnectivity of self-referential midline regions) and aberrant SN-FPN (frontoparietal network) interactions	Fragmented affective coherence and self-concept (from atypical self-referential processing); compromised social decoding (e.g., misperception of intentions of others, theory-of-mind failures); and embodied regulation disruptions (hallucinations, delusions of control suggesting self-other boundary confusion)
Major depressive disorder (MDD)	DMN hyperactivity and failure to deactivate during tasks; weaker coupling of DMN with FPN (executive control network)	Overactive self-referential processing hurts emotional regulation (constant negative thoughts about the self); decreased integrated state of body and mind control of attention (cannot move away from internal thoughts thanks to DMN activation), resulting in isolation; social perception distortions (negative self-bias when receiving social feedback)
Post-traumatic stress disorder (PTSD)	Overactive SN (hypervigilance circuitry) coupled with hypoactive DMN and FPN (impaired default-mode and executive function connectivity)	Affective disruptions (traumatic memories intrude and hyperarousal disrupt the integrated sense of self-narrative); social challenges (warped social perceptions where people cannot be trusted or social cues are misread based on trauma response); problems with embodied agency (dissociating and feeling alien to one’s body based on trauma stimuli)
Attention-deficit/hyperactivity disorder (ADHD)	Ineffective SN-mediated toggling between DMN and FPN; lack of DMN deactivation (overactive mind-wandering)	Embodied control deficits (inattention/impulsivity shows inability to modulate attention and behaviors—unable to switch out of self-referential DMN “daydreaming” mode); mild social decoding challenges (impulsive reactions prevent awareness of social signals); overwhelming internal distractibility prevents prolonged task-oriented self-activities.
Borderline personality disorder (BPD)	Anomalous DMN connectivity (fluctuations in self-referential network functioning); inconsistent SN functioning (hyperactive insula/ACC related to emotional awareness)	Unpredictable emotional consistency (frequent changes in mood and problematic sense of self-identity fragmentation due to dysregulated integration of emotions into self); compromised attunement and perspective taking (difficulty comprehending others’ motivations or feelings, frequently misjudging social signals); dysregulated embodied agency behaviors that signal failures of mindfully living in one’s body
Alzheimer’s disease (AD)	DMN degeneration (posterior cingulate and hippocampal network hypometabolism and connectivity loss); progressive cortical atrophy extending to FPN in later stages	Fragmented episodic memory and emotional stability (deteriorating life story and emotional consistency); diminished social apprehension (difficulty recognizing once-familiar people or social scenarios as the disease progresses); diminished self-directed agency (confusion, inability to map body cues to spatial awareness in later stages)
Frontotemporal dementia (bvFTD)	Salience network atrophy (fronto-insular and anterior cingulate degeneration); orbitofrontal network dysfunction	Decreased social awareness [severe empathic failures and inappropriate social behavior due to insular–ACC degeneration related to socio-emotional understanding; apathy or excessive impulsive emotion due to frontolimbic involvement; basic sensorimotor abilities retained but embodied regulation of social conduct compromised (disinhibition)]

Summary of how large-scale network abnormalities in selected disorders correspond to disruptions in DSR components. DMN, default mode network; SN, salience network; FPN, frontoparietal network. DSR components include *affective coherence* (integration and consistency of one’s emotional state with self-representation), *social decoding* (accurate interpretation of social signals and theory-of-mind processes), and *embodied control* (sense of agency and regulation of bodily self in the environment). Each disorder tends to perturb specific networks, leading to characteristic breakdowns in one or more of these self-representation capacities.

## 5 Similarities across disorders and next steps, network-level impairment as a unifying principle

Although all these disorders have distinct etiologies and clinical presentations, they have been aligned under one review. The unifying component is that they have all been shown to exist along the lines of large-scale brain networks championing the “default spatial representation.” The problem is taking an integrated, network perspective and rendering it clinically useful. Our unifying framework reveals that the DSR does not reside in one area but emerges from the dynamics of multiple simultaneously recruited networks like the DMN, dorsal attention network, ventral attention network, and salience network (Seeley et al., 2007). How one’s self-in-space can become “faded” is likely due to a lack of integration within this widely distributed system (Cauda et al., 2011; Northoff and Sibille, 2014; Van Dijk et al., 2010).

Mechanistic insights: Next steps must adhere to more precision in mechanistic insights. For example, where might disturbance of excitatory/inhibitory neurotransmission dynamically across manifestations contribute to the integrity/breakdown of the DSR? How might disrupted oscillatory dynamics—in the form of atypical alpha oscillations in neglect (Klimesch, 2012), gamma/theta coupling/disorganization in disorders of consciousness (Fries, 2015; Singer, 1999), or breakdowns in long-range white matter connectivity—contribute to disruptions of the DSR (Deco et al., 2015; Grefkes and Fink, 2011)? Furthermore, how might interoception play a role in continuously assessing micro-levels of this spatial self (updated self-DSR) only to ground it/facilitate integrity at a larger scale (Seth and Friston, 2016; Seth, 2013; Jerath et al., 2015)?

Diagnostic/prognostic biomarkers: Diagnostics across many of these disorders rely on behavioral assessments with subjectivity and limitations—a serious drawback with non-responsive patients. Prognosis stems from uncertainty. Instead, DMN connectivity measures, DMN–attention network coupling, or other information evaluating DSR integrity might signify diagnostic/prognostic biomarkers; currently, research is reviewing how DMN functional connectivity may predict recovery from DOCs (Martini et al., 2019), differentiate types of neglect, or assess those at greatest risk for dementia—with subsequent development of reliable DSR diagnostic markers via resting-state fMRI, EEG coherence, or graph theory analyses in clinical application for all future directions (Sestieri et al., 2017).

## 6 Therapeutic interventions

Currently, many rehabilitation approaches are compensatory rather than restorative and have little effect. The goal of our framework is to shift toward compensatory network-level interventions that also have the potential to restore integrity and self-calibration of the DSR directly.

Neuromodulation: Techniques such as transcranial magnetic stimulation (TMS) and transcranial direct current stimulation (tDCS) can rebalance interhemispheric inhibition or upregulate/downregulate activity in relevant DMN/attention network nodes; deep brain stimulation (DBS) is under evaluation for severe DOCs.

Neurofeedback: Teaching patients how to self-regulate DMN activity or DMN-attention network coupling via real-time fMRI or EEG neurofeedback is a promising, non-invasive technique.

Pharmacology: An appeal for certain drugs that target neurotransmitter systems (e.g., cholinergic, dopaminergic) specifically known to modulate network dynamics and attention could serve to restore DSR functioning.

Rehabilitation: Existing therapies (e.g., prism adaptation for neglect and virtual reality navigation training for spatial disorientation) could be adapted to specifically facilitate restoration of the DSR by reinforcing reintegration of neglected space or recalibrating internal spatial representation.

## 7 Computational modeling

At present, we lack a precise, quantitative sense of how the DSR emerges and disintegrates. We advocate the need for advanced computational generative models to simulate the emergence and maintenance of a “default spatial representation” in a networked architecture (Friston and Kiebel, 2009) to simulate specifics of lesions or networked dysfunctions that lead to “fading” of such representations or dis-coherence, which can then simulate clinical symptoms observed (Hohwy, 2013); to simulate potential outcomes of neuromodulation, which can be quantitatively assessed as a reference for therapeutic adjustments (Seth, 2013, 2014; Friston and Kiebel, 2009; Seth and Friston, 2016). Bridging Scales: Ultimately, all this comes down to the challenge of bridging molecular and cellular findings (e.g., what populations are firing, what synaptic plasticity mechanisms exist) with networked scales and topics on behavior. If we can understand the mechanism by which molecular deficit contributions lead to the informed DSR deviance, then we can facilitate true interventions.

## 8 Conclusion

**Figure 1:**
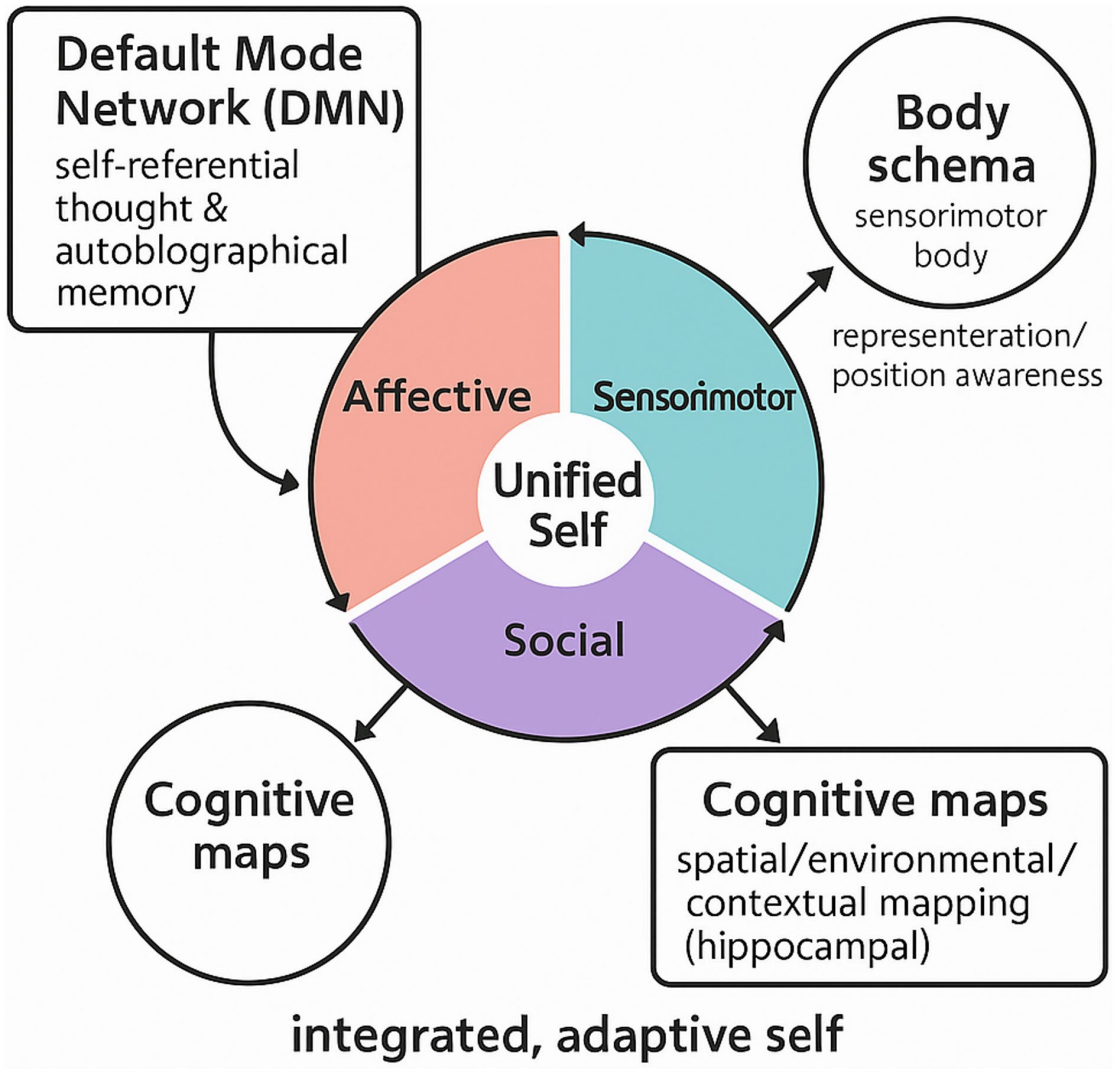
Conceptual illustration of the dynamic self-representation (DSR) framework contrasted with related frameworks.

Restoring the spatial self: Our ability to access one continuous, coherent “default spatial representation” is central to who we are and how we experience the world. As argued within this article, many seemingly diverse insults to the mind—from the striking unilateral deficit associated with neglect to the profound disembodiment challenges in disorders of consciousness—all link back to one major overlapping dysfunctional pathway, an interference with default internal spatial representation. By adopting such a unifying framework, the observations extend beyond merely classification inclusions; it provides clarity to shared underlying networked dysfunctions. Thus, this truly novel perspective will not only enhance theoretical understanding of life-changing conditions but also lead to clinical diagnostic biomarkers and networked intervention opportunities. Ultimately, helping people reclaim their sense of self-in-space will improve quality of life and functional independence from these otherwise complicated neurologically manifesting realities. Future directions for the dynamic self-representation model involve various testable hypotheses and possibilities for empirical investigation. For instance, if independent but coupled DSR processes increase self-functionality, then positive or negative manipulation/resuscitation of DMN/SN/FPN network coordination (e.g., through therapy, neuromodulation, and training) should predict corresponding functional units of self in measurable quantities; this could be measured through longitudinal neuroimaging studies that correlate integrated DMN/SN/FPN networks with longitudinal evolution of subjective report or behavioral change in typical functionality. Cross-diagnostic investigations may further lead to prediction checks—for instance, assessing whether specific DSR disruptions (i.e., failure of affective–social network coupling, deviant self–other representation) are present in a variety of disorders that, when they emerge by themselves based on coupling failures, could posit such factors as transdiagnostic biomarkers reflecting dysfunctional self-representation derived from DSR (see Figure 1). This also provides interesting avenues for developmental research; if DSR is the ultimate factor that transcends representation, then one can predict that the independent factors will emerge and gradually come together during childhood and early adolescence based on brain maturation, as trajectories differ for those ultimately vulnerable to diverse disorder types. For example, if affective coherence networks emerge earlier in resolution during early adolescence than others, one can predict that this cohort will have a more resilient self-concept. If individuals can relieve compulsive symptoms in one realm (say, through social decoding training), and the findings suggest neuro-connection change in other realms, as well as DMN/SN relationships, then these hypotheses can increasingly validate the DSR model thereafter. Longitudinal studies must confirm or deny such empirical hypotheses, as should cross-sectional and cross-diagnostic studies. Successes along these paths will not only affirm DSR’s ability to explain but also provide the foundation for new treatment options—therapeutic approaches using network-based neuromodulation or integrative psychotherapy targeting an intersection of conditions offered by a better understanding of multi-dimensional manifestations rather than singular disassociations will encourage results directed toward restoration of appropriate dynamic balance for self across affective, embodied, and social realms. Ultimately, this conversion from theoretical model to real-world application is necessary to understand the self and disturbances to it, even further, to streamline the treatment of disturbances that plague it.

## Data Availability

The original contributions presented in the study are included in the article/supplementary material, and further inquiries can be directed to the corresponding author.
